# Sprouty1 regulates gonadal white adipose tissue growth through a PDGFRα/β-Akt pathway

**DOI:** 10.1080/21623945.2021.1987634

**Published:** 2021-10-29

**Authors:** Xuehui Yang, Shivangi Pande, Robert A. Koza, Robert Friesel

**Affiliations:** a Center for Molecular Medicine, Maine Medical Center Research Institute, Scarborough, ME, USA; b Graduate School of Biomedical Sciences and Engineering, University of Maine, Orono, ME, USA

**Keywords:** Adipocyte, Sprouty1, proliferation, differentiation, fibrosis, cell signalling

## Abstract

Expansion of visceral white adipose tissue (vWAT) occurs in response to nutrient excess, and is a risk factor for metabolic disease. SPRY1, a feedback inhibitor of receptor tyrosine kinase (RTK) signaling, is expressed in PDGFRa+ adipocyte progenitor cells (APC) in vivo. Global deficiency of *Spry1* in mice results in disproportionate postnatal growth of gonadal WAT (gWAT), while iWAT and BAT were similar in size between *Spry1*KO and WT mice. *Spry1* deficiency increased the number of PDGFRa+ stromal vascular fraction (SVF) cells in gWAT and showed increased proliferation and fibrosis. *Spry1*KO gWAT had increased collagen deposition and elevated expression of markers of inflammation. In vitro, SPRY1 was transiently down regulated during early adipocyte differentiation of SVF cells, with levels increasing at later stages of differentiation. SPRY1 deficiency enhances PDGF-AA and PDGF-BB induced proliferation of SVF cells. Increased proliferation of SVF from *Spry1*KO gWAT accompanies an increase in AKT activation. PDGF-AA stimulated a transient down regulation of SPRY1 in wild type SVF, whereas PDGF-BB stimulated a sustained down regulation of SPRY1 in wild type SVF. Collectively, our data suggest that SPRY1 is critical for regulating postnatal growth of gWAT by restraining APC proliferation and differentiation in part by regulation of PDGFRa/b-AKT signaling.

## 1. Introduction

White adipose tissue (WAT) is a highly dynamic organ that coordinates metabolic homoeostasis by expanding upon nutrient excess, and releasing lipids in response to energy demand. WAT expands either through adipocyte hypertrophy or hyperplasia, and this expansion requires remodelling of the adipose tissue microenvironment including the extracellular matrix (ECM) [1,2]. The ECM also plays a role in APC differentiation, and each WAT depot has a distinct origin during mouse development [3]. Dysregulated ECM remodelling in WAT results in fibrosis, which is often seen as a pathological consequence of the obese state [2]. Despite a large body of research, the mechanisms leading to WAT fibrosis and its subsequent effects on obesity-associated metabolic dysfunction remains poorly understood.

The expansion of WAT occurs through either adipocyte hypertrophy (increase in cell size) or adipocyte hyperplasia (increase in cell number). Chronic adipocyte hypertrophy is accompanied by changes in adipokine secretion, inflammation, fibrosis and cell death, whereas adipocyte hyperplasia results in an increased number of adipocytes but with less inflammation and fibrosis [4]. Adipocyte hyperplasia is commonly associated with metabolically healthy obese individuals, whereas adipocyte hypertrophy is more often associated with obese individuals with metabolic syndrome. APC are maintained in the interstitial space between mature adipocytes and differentiate into adipocytes to meet demand for energy storage. The molecular mechanisms regulating APC maintenance and differentiation remain largely unknown.

Adult APCs are comprised of a highly heterogeneous population of cells [4]. Accumulating evidence indicates that platelet-derived growth factor receptor alpha (PDGFRα), is a critical functional marker of APC [5,6]. PDGFRβ is also expressed in APC, however evidence suggests that PDGFRα expression precedes that of PDGFRβ in a subset of visceral WAT (vWAT) [7,8]. PDGFRs are RTKs that are activated upon stimulation with ligand, and play important roles in normal development, as well as many disease processes [9]. PDGFRα has been shown to control the balance of stromal and adipogenic cells, and activation of PDGFRα and PDGFRβ cell-autonomously inhibits embryonic adipocyte differentiation in vivo [6]. On other hand, it was shown that PDGF-AA/PDGFRα/PI3K-AKT signalling maintains APC residing in the dermis; and, deficiency of PDGFRα results in depletion of dermal APC and loss of dermal WAT during hair cycles [10]. PDGFRs are also involved in adipose remodelling and dysfunction. PDGF-BB/PDGFRβ signalling is associated with obesity and metabolic dysfunction [11], and PDGFRα activation was shown to switch APC plasticity towards a profibrotic phenotype, and led to obesity-induced WAT fibrosis [12,13]. Together, these data indicate that PDGF-PDGFRα/β signalling is critical to WAT development, homoeostasis, and dysfunction.

SPRY1 is a feedback modulator of RTK signalling RTK signalling, particularly in development and tissue regeneration by epidermal growth factor (EGF), fibroblast growth factors (FGF), platelet-derived growth factors (PDGF), erythropoietin and glial cell-derived neurotrophic factor (GDNF) pathways [14–16]. SPRY1 also plays an important role in adipocyte differentiation. Previously, we showed that SPRY1 favours osteoblastogenesis of bone marrow mesenchymal cells at the expense of adipogenesis [17]. Recently, it was reported that SPRY1 is a weight loss target gene whose expression increases in APC after weight loss interventions in humans [18], however the mechanism by which SPRY1 regulates APC function is not completely understood.

The diversity and broad distribution of WAT in the mouse is challenging for conditional gene targeting strategies in part due to different developmental origins of different fat depots. Therefore, we used a global *Spry1* deficient mouse model (*Spry1*KO) to study the role of SPRY1 in WAT growth. We found that SPRY1 was expressed in PDGFRα expressing SVF in vivo, and a deficiency of SPRY1 selectively led to disproportionate post-natal growth of gWAT and rWAT. The SPRY1 deficient mice also developed fibrosis of gWAT upon ageing. This fibrosis was accompanied by an increase in expression of markers of proliferation and inflammation. We show that in wild type primary adipogenic cells that SPRY1 is transiently down regulated during adipocyte differentiation and robustly expressed upon late stages of adipocyte differentiation. Mechanistically, loss of SPRY1 enhanced AKT activation by PDGF-AA and PDGF-BB in stromal vascular fraction (SVF) cells, and that pretreatment of wild type SVF with PDGF-BB inhibited adipocyte differentiation, whereas PDGF-AA was without effect. These results suggest a critical role for SPRY1 in SVF growth and differentiation at least in part by modulating PDGFRα/β-AKT signalling.

## 2. Results

### 2.1. Spry1 deficiency promotes gonadal white adipose tissue expansion and fibrosis upon ageing

**Figure 1.**
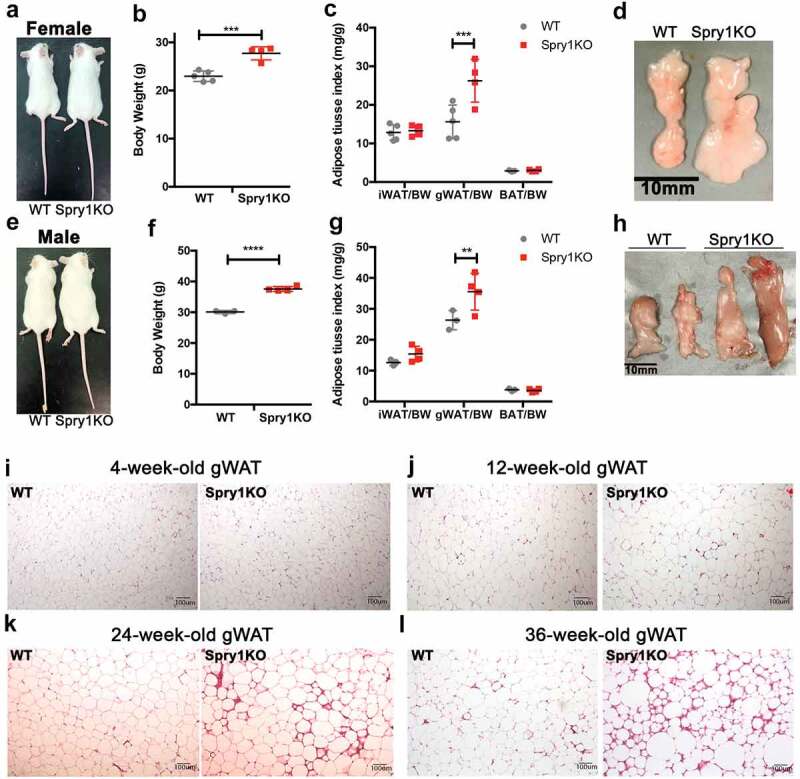
Loss of *Spry1* promotes postnatal gonadal white adipose tissue growth. Phenotyping of *Spry1KO* deficiency mice shows that 12-week-old female mice are heavier (a, b) and have an increased gWAT to body weight (BW) ratio compared to WT mice (c, d) (WT; n = 5, *Spry1*KO; n = 4). Similarly, 12-week-old male *Spry1*KO mice are heavier (e, f) and also have an increased gWAT/BW ratio (g, h) (WT; n = 3, *Spry1*KO; n = 4). i-l: H&E staining of male gWAT shows progressive fibrosis with ageing in *Spry1*KO mice compared to WT mice; i) gWAT tissues from mice at 4-weeks-of-age, j) 12- weeks-of-age, k) 24-week-of-age, and l) 36-weeks-of-age. **: p < 0.01; ***: p < 0.001; ****: p < 0.0001 (unpaired t-test or 2way-ANOVA analysis followed by multiple comparison)

Mice with global deficiency of *Spry1* on a C57BL/6 background do not survive postnatally due to supernumerary ureteric buds [15]. However, *Spry1* deficient mice on an FVB background survive postnatally with incomplete penetrance of unilateral hydronephrosis in males (~20%) and enlarged uterus in females (~70%). These mice survive well into adulthood. Mice that showed these defects were excluded from this study. During the course of our phenotypic analysis, we observed that both male and female *Spry1KO* mice weighed more than their wild type littermates at twelve weeks-of-age (Figure 1(a,b,e,f)). Analysis of major fat depots showed that male and female *Spry1*KO mice had larger gWAT depots than WT littermates, but with no significant difference in inguinal subcutaneous WAT (iWAT) and brown adipose tissue (BAT) (Figure 1(c,d,g,h)). We also collected and measured retroperitoneal WAT (rWAT), and found a significant increase in rWAT/BW index (mg/g) (WT vs. *Spry1*KO: 5.803 ± 0.229 vs. 8.795 ± 0.5785, p < 0.01). We next, performed a detailed histological analysis on gWAT from *Spry1*KO and WT mice at various ages. At four and twelve weeks-of-age, gWAT from *Spry1*KO and WT mice was similar in appearance (Figure 1(i,j)) in size and morphology. However, at 24 weeks-of-age *Spry1*KO gWAT showed a fibrotic appearance that worsened by 36 weeks-of-age (Figure 1(k,l)). This fibrosis was most pronounced at the tips of the gWAT. Together, these data show that global deficiency of *Spry1* results in increased body weight, in part due to increased gWAT expansion, and that gWAT of *Spry1*KO mice becomes increasingly fibrotic with age.

### 2.2. gWAT from Spry1KO mice show increased extracellular matrix deposition and markers of inflammation

**Figure 2.**
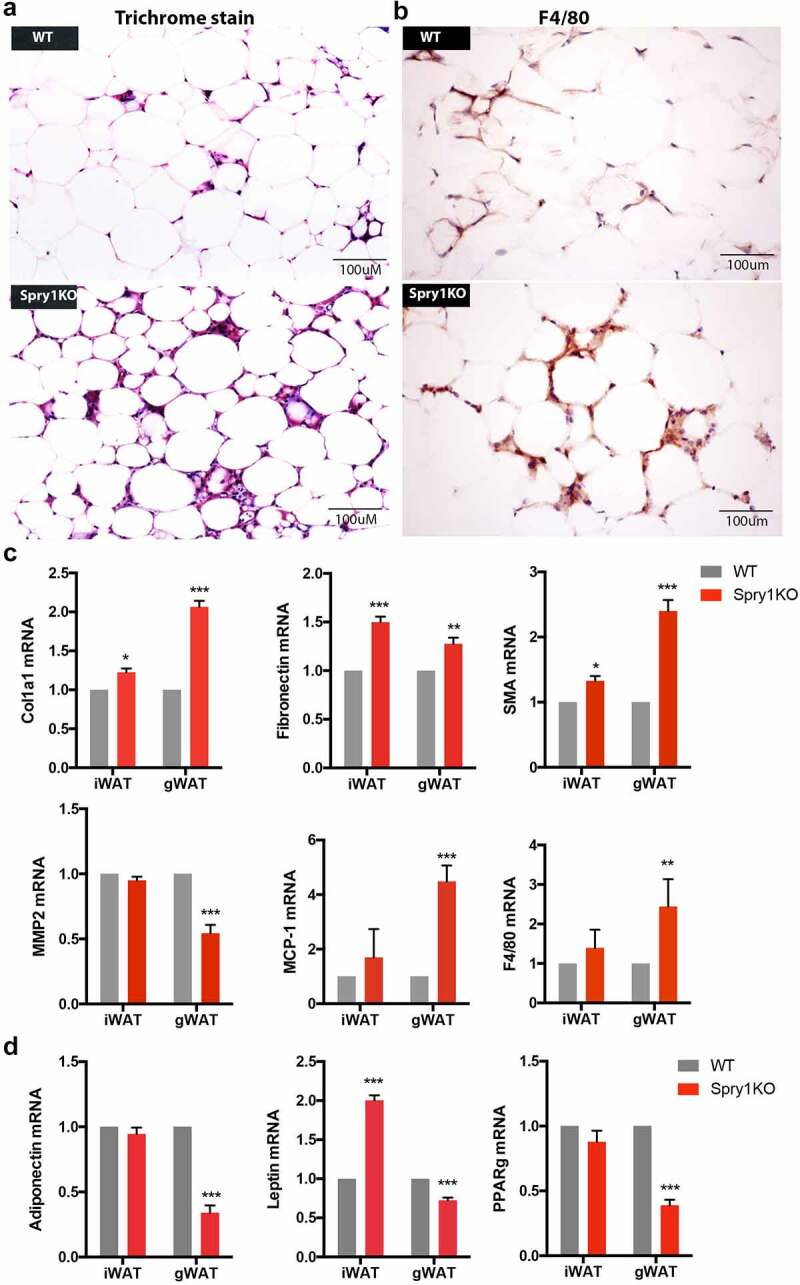
Loss of *Spry1* increases gWAT fibrosis and inflammation with ageing. a) Sections of gWAT from thirty-six-week-old Spry1KO or WT mice were stained with Trichrome to visualize collagen content. b) Immunohistochemistry staining with F4/80 antibody to visualize macrophage-like cells. c) RT-qPCR analyses of mRNA from iWAT and gWAT from male 36-week-old WT (n = 4) and *Spry1KO* (n = 4) mice. The results show that loss of *Spry1* significantly increased *Col1a1, Fn1, Sma, Mcp-1* and *F4/80* mRNA expression in gWAT, but decreased *Mmp2* expression. Loss of *Spry1* also increased *Col1a1* and *SMA* mRNA expression in iWAT. d) RT-qPCR analyses of expression of adipogenic markers. The results show that loss of *Spry1* significantly decreased *Adiponectin* (ADPN), *PPARγ*, and increased *leptin* mRNA expression. *: p < 0.05; **: p < 0.001; ****: p < 0.0001 (2way ANOVA followed by multiple comparison)

Fibrotic and inflamed adipose tissue typically shows crown-like structures in the spaces between adipocytes and are characterized by increased collagen deposition and cytokine expression [2,4,6]. Trichrome staining of *Spry1*KO gWAT at 36 weeks-of-age showed increased deposition of collagen in the crown-like structures when compared to gWAT of WT mice of the same age (Figure 2(a)). Immunostaining with the macrophage marker *Adgre1* (F4/80) showed increased staining in the crown-like structures of *Spry1*KO compared to WT littermates (Figure 2(b)). RT-qPCR analysis showed an increase in the expression of *Col1a1* and *Fn1* (fibronectin) mRNA in *Spry1*KO gWAT and an increase in the expression of the fibroblastic marker *Acta2* (SMA) relative to WT gWAT (Figure 2(c)). Interestingly, iWAT from *Spry1*KO mice showed a small but significant increase in these markers as well. We also measured markers of inflammation. We observed that the matrix metalloproteinase *Mmp2* was decreased in expression in *Spry1*KO gWAT compared to WT gWAT. Expression of mRNA for the inflammatory cytokine *Ccl2* (MCP1) and the macrophage marker F4/80 were also increased in *Spry1*KO gWAT. There were no significant increases in markers of inflammation in iWAT from either *Spry1*KO and WT mice. Our RT-qPCR results also show that adiponectin, *Lep* (leptin), and *Pparg* expression was decreased in *Spry1*KO gWAT, whereas adiponectin and *Pparg* were unchanged in iWAT. Interestingly, *Lep* was increased in iWAT of *Spry1*KO mice (Figure 2(d)). These results indicate that the increase in crown-like structures and fibrosis in *Spry1*KO gWAT is due in part to increased deposition of extracellular matrix and increased expression of markers of inflammation.

### 2.3. Spry1 is expressed predominantly in the stromal vascular fraction of adipose tissue

**Figure 3.**
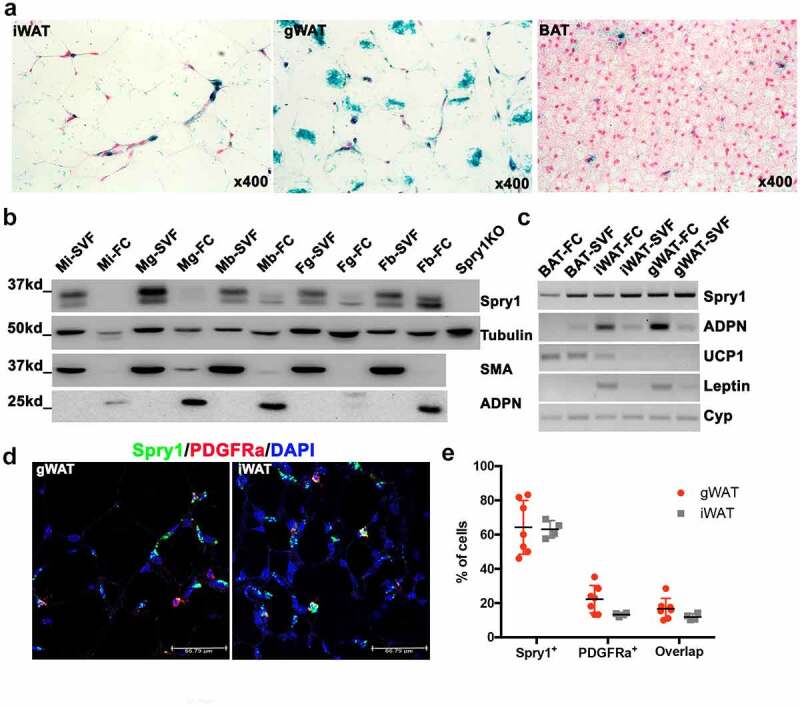
*Spry1* is expressed in adipocytes and the stromal vascular fraction, and SPRY1 protein is down regulated upon induction of adipogenic differentiation. a) Whole mount X-gal-stained adipose tissue sections from 8-week-old *Spry1KO* mice that have *LacZ* gene insertion under control of the *Spry1* gene promoter. b) Immunoblot analysis of mature adipocytes and SVF with SPRY1 and ADPN antibodies shows that SPRY1 is expressed in iWAT and gWAT with relatively higher expression in the SVF, and lower in mature adipocytes (fatty cake). c) RT-PCR analyses shows *Spry1* mRNA expression in SVF and mature adipocytes. d) Representative images of RNAScope analysis on gWAT and iWAT using probes for *Spry1* and *Pdgfrα* showing partial overlap in their expression. e) Quantification of the percentage of *Spry1* or *Pdgfα* mRNA positive cells per field of *Spry1* and *Pdgfα* co-expression cells (3–5 fields/section, 7 sections from 3 mice for gWAT; and 3–5 fields/section, 5 sections from 3 mice for iWAT). Note: male iWAT SVF (Mi-SVF); male iWAT fatty cake (mi-FC); male gWAT SVF (Mg-SVF); male gWAT fatty cake ((Mg-FC); male BAT SVF (Mb-SVF); male BAT fatty cake (Mb-FC); female gWAT SVF (Fg-SVF); female gWAT fatty cake (Fg-FC); female BAT SVF (Fb-SVF); female BAT fatty cake (Fb-FC)

The adipose tissue stromal vascular fraction (SVF) is the niche where APCs reside. In adult mice, a population of PDGFRα+ cells in the SVF give rise to mature adipocytes in adult mice [1,4]. To further investigate the role of SPRY1 in adipocyte growth and differentiation, we used mice with a β-galactosidase reporter gene inserted into the *Spry1* locus and driven by the *Spry1* promoter [15]. X-gal staining of adipose tissue sections of eight-week-old mice showed robust staining in gWAT with less X-gal staining seen in iWAT, and most of this staining appeared in the SVF (Figure 3(a)). A small amount of staining was observed in brown adipose tissues (Figure 3(a)). We also performed immunoblotting on mature adipocytes and SVF cells isolated by centrifugation. SPRY1 is highly expressed in the SVF of iWAT, gWAT, and BAT, which co-expressed SMA, a marker of mural/adult APCs in WAT (Figure 3(b)) [19,20]. SPRY1 was expressed at lower levels in mature adipocytes which were identified by blotting for adiponectin. Similar expression patterns were observed by RT-PCR (Figure 3(c)). PDGFRα is a marker for mesenchymal cells in adult mice, and Lin- PDGFRα+ SVF cells are enriched with APC [21]. We used RNAscope to determine the spatial distribution *Spry1* and *Pdgfra* in gWAT and iWAT because SPRY1 functions in most contexts as a feedback inhibitor of receptor tyrosine kinase signalling such as PDGFR [14]. *Spry1* mRNA was detected in a majority of gWAT and iWAT SVF cells, however, *Pdgfra* was detected in only 15–20% of SVF cells, and most *Pdgfra*+ cells also expressed *Spry1* (Figure 3(d,e)).

### 2.4. SPRY1 is dynamically expressed during adipocyte differentiation, and loss of Spry1 promotes SVF adipocyte differentiation

**Figure 4.**
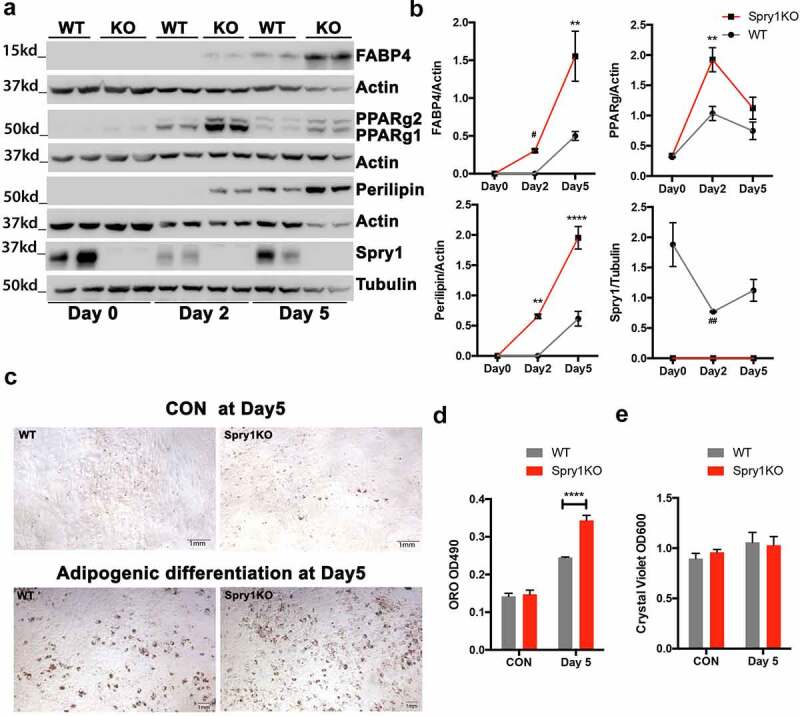
SPRY1 is dynamically expressed during adipocyte differentiation, and loss of Spry1 enhances SVF cell adipogenic differentiation. a) Immunoblot analysis of a time-course of adipogenic differentiation using SVF cells from WT and *Spry1*KO iWAT at passage 1. b) Quantification of the protein level of PPARγ, FABP4, Perilipin, and SPRY1 from immunoblotting experiments in A. c) Representative images of Oil Red O staining at day 5 post adipogenic differentiation. d) Quantification of oil Red O staining 5 days after induction of adipogenic differentiation (n = 4 per group). Statistics are from at least three replicates. e) After extraction of oil Red O dye from D, cells were stained with crystal violet for relative cellular content. **: p < 0.01; ****: p < 0.0001(2way ANOVA followed by multiple comparison)

To determine the expression of Spry1 in SVF during adipocyte differentiation we isolated SVF from iWAT, which maintains adipogenic potential in culture better than gWAT. SVF cells were isolated from WT and *Spry1*KO mice, put into culture and induced to differentiate for 5 days as described in materials and methods. We observed that in WT SVF, SPRY1 is highly expressed before induction of differentiation, and declines significantly after 2 days of differentiation, and expression begins to increase 5 days after induction of differentiation (Figure 4(a,b)). We also observed that PPARγ levels were significantly higher in *Spry1*KO SVF than in WT 2 days after induction of differentiation (Figure 4(a,b)). In addition, PLIN protein levels were higher in *Spry1*KO SVF versus WT. Similarly, FABP4 protein was more highly expressed in *Spry1*KO SVF compared to control. Lastly, SVF from *Spry1*KO mice showed significantly more oil red O staining after 5 days of differentiation compared to WT SVF with little significant difference in cell number as determined by crystal violet staining (Figure 4(c–e)).

### 2.5. SPRY1 deficiency increases proliferation of SVF cells in vivo and in vitro

**Figure 5.**
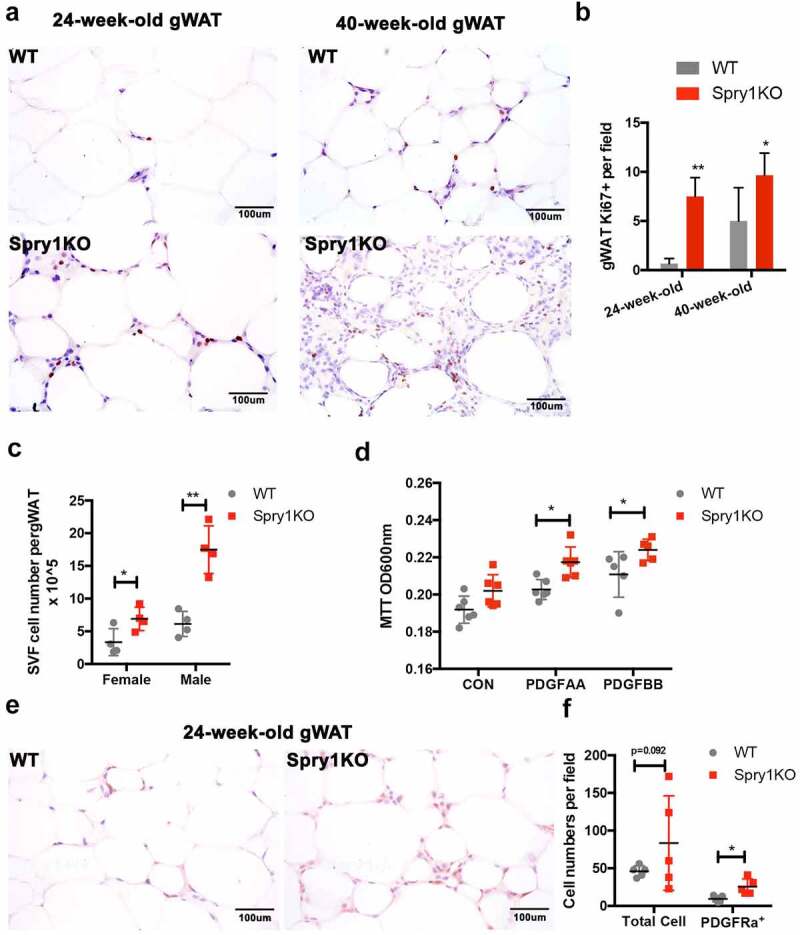
Loss of *Spry1* increases adipose stromal vascular fraction cell proliferation both in vitro and in vivo. a) Immunohistochemically staining with Ki67 antibody on gWAT sections from 24- or 40-week-old WT and *Spry1*KO males. Representative images showed more Ki67 positive cells in *Spry1*KO gWAT compared to that in WT; b) Quantification of Ki67 positive cells per field shows a significant increase of Ki67 positive cells in *Spry1*KO gWAT (24-week-old, n = 5 for each genotype; 40-week-old, n = 4 for each genotype). c) Cell counting of collagenase fractionated gWAT SVF cells from 12-week-old WT and *Spry1*KO mice (n = 4 for each genotype). The results show that loss of *Spry1* significantly increased the number of SVF cells in gWAT. d) MTT assays show that loss of *Spry1* significantly increased PDGF-AA and PDGF-BB mediated SVF cell proliferation in vitro (representative of 3 experiments, n = 5 per condition). e) Representative images of immunohistochemically staining of PDGFRα on gWAT sections from 24-week-old mice. f) Quantification of PDGFRα+ cells show an increase of the number of PDGFRα+ cells per field in *Spry1*KO gWAT compared to that in WT controls (n = 5 for each genotype). *: p < 0.05; **: p < 0.01 (2way-ANOVA followed by multiple comparison)

To evaluate whether the increased size of gWAT in *Spry1*KO mice was due to increased proliferation, we performed immunohistochemical staining of sections of gWAT from 24- and 36-week-old *Spry1*KO and WT mice. *Spry1*KO gWAT showed an increase in the number of Ki67+ cells compared to WT gWAT (Figure 5(a,b)). In addition, gWAT SVF cell number was greater in both male and female mice at 12-weeks-of age (Figure 5(c)), which is consist with the enlarged gWAT tissues. Finally, SVF isolated from gWAT of *Spry1*KO and WT mice showed increased proliferation in response to PDGF-AA and PDGF-BB in MTT assays (Figure 5(d)). Because PDGFRα is a marker for APCs, we performed immunohistochemistry on sections of gWAT from 24-week-old *Spry1KO* and WT mice. There are more SVF cells per field in *Spry1*KO gWAT than that in WT mice but this difference did not achieve statistical significance (Figure 5(e,f)). There were however, significantly more PDGFRα+ SVF cells in sections of *Spry1*KO gWAT than in WT gWAT (Figure 5(f)). Together, these data indicate that *Spry1* deficiency in gWAT results in increased proliferation of SVF cells and this may be due in part to an increased in the number of PDGFRα+ SVF cells.

### 2.6. PDGF down regulates SPRY1 expression in SVF and Spry1 deficiency enhances AKT activation

**Figure 6.**
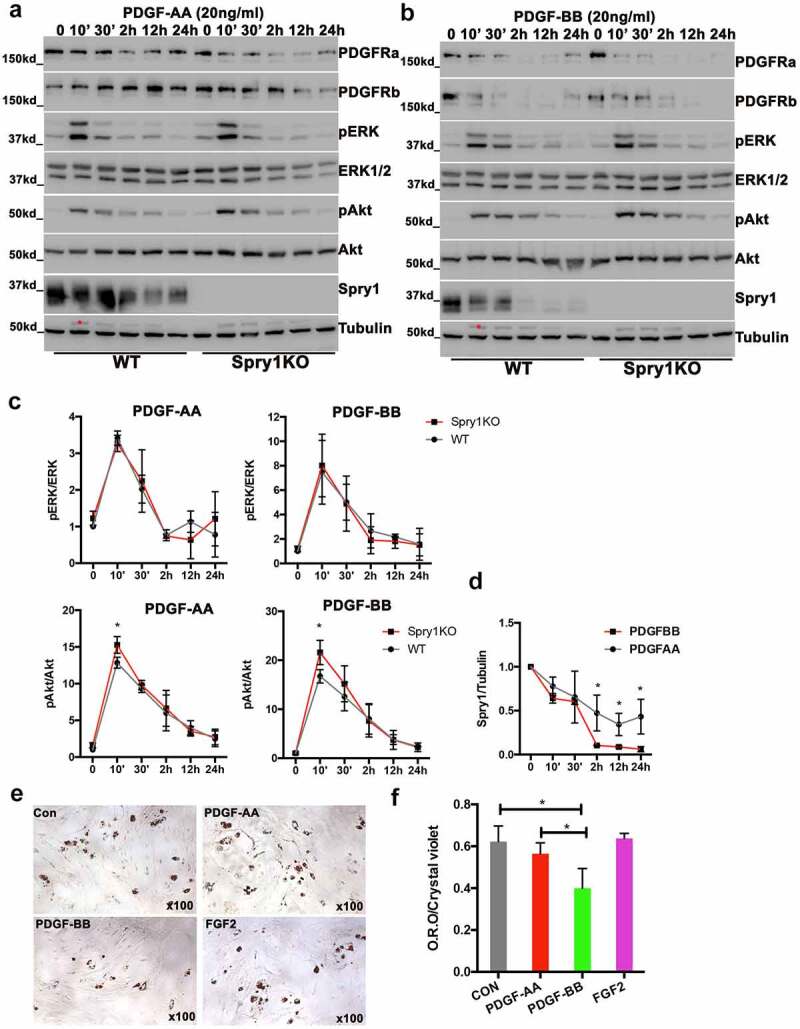
PDGF down regulates SPRY1 expression in SVF and loss of *Spry1* enhances Akt activation. a, b) Immunoblot analysis of a time-course of PDGF-AA or PDGF-BB stimulated SVF cells from *Spry1KO* and wild type mice gWAT. c) Quantification of pERK/ERK and pAKT/AKT from three time-course experiments shows that loss of *Spry1* enhanced PDGF stimulated peak pAKT activation, but had no effect on pERK activation. d) Quantification of SPRY1 protein shows that PDGF-AA and PDGF-BB stimulation differentially down regulated SPRY1 in gWAT SVF cells ex vivo. SPRY1 protein was markedly down-regulated by PDGF-BB, whereas it was modestly and transiently down regulated by PDGF-AA (the data are from 3 independent experiments). e) Representative Oil Red O staining of wild type SVF pretreated with PDGF-AA, PDGF-BB or FGF2 followed by adipogenic differentiation for 8 days. f) Quantification of Oil Red O staining from one of three 4–6experiments. The results show that PDGF-BB but not PDGF-AA pre-treatment significantly decreased the adipogenic differentiation of SVF cells as indicated by Oil Red O staining (the data are from one of 3 independent experiments with 4–6 replicates). *: p < 0.05, (2way-ANOVA analysis followed by multiple comparison)

PDGFR signalling regulates APC development and plays a role in stromal versus adipocyte differentiation [12,13]. Because SPRY1 is a regulator of RTK signalling, we investigated whether responses to PDGF stimulation were altered in SVF cells due to *Spry1* deficiency. SVF cells from *Spry1*KO and WT mice gWAT were used at passage one for time course experiments in response to either PDGF-AA or PDGF-BB. Interestingly, *Spry1* deficiency had no effect on ERK activation by either PDGF-AA or PDGF-BB (Figure 6(a–c)). However, *Spry1* deficiency did enhance peak activation of AKT by both PDGF-AA and PDGF-BB. In addition, we noted that in wild type SVF cells, PDGF-AA resulted in a transient down regulation of SPRY1, whereas PDGF-BB induced a sustained down regulation of SPRY1 (Figure 6(e)). Based on the differential down regulation of SPRY1 by PDGF-AA and PDGF-BB we asked whether the presence PDGF-AA or PDGF-BB affected adipocyte differentiation of wild type SVF. Pretreatment of wild type SVF with PDGF-AA, PDGF-BB or FGF2 for 24 h showed that PDGF-BB but not PDGF-AA or FGF2 inhibited adipocyte differentiation as determined by oil red O staining (Figure 6(f,g)). Together, these data suggest that PDGF-BB down regulates SPRY1 expression, and inhibits adipocyte differentiation.

## 3. Discussion

Our studies show that *Spry1* deficiency promoted the disproportionate growth of gWAT in both male and female mice. Male and female *Spry1*KO mice showed enlargement of gWAT at twelve weeks of age while iWAT was similar in size between *Spry1*KO and WT mice when normalized to body weight. The fact that the gWAT depot was disproportionately larger in *Spry1*KO mice in the absence of nutrient excess, suggests that this may be due to the developmental in origin. Indeed, lineage tracing studies indicate that iWAT is derived from the posterior lateral plate mesoderm, whereas gWAT is derived from somatic mesoderm, and the APCs that give rise to these depots have distinct expression profiles [5]. These lineage tracing studies also demonstrated a gradient of somite-derived APC along the anterior-posterior axis of gWAT, with the anterior most portion referred to as the tip showing the highest percentage of somite-derived APC [5]. This is also the region of gWAT that showed the greatest extent of fibrosis in older mice in our studies. Our data suggest that SPRY1 has a unique role in regulating the postnatal growth of gWAT and that *Spry1* deficiency results dysregulated proliferation and differentiation of gWAT.

Our results show that as *Spry1*KO mice age, their gWAT become progressively more fibrotic in appearance with increased deposition of ECM and the expression of markers of inflammation. This increase in fibrosis in *Spry1* deficient mice is also accompanied by an increase in markers of proliferation, and an increase in the number of PDGFRα+ SVF cells. In SVF from WT mice, SPRY1 is more widely expressed than PDGFRα, however, most PDGFRα expressing cells also expressed SPRY1. Recent studies show that the activating mutation in *PDGFRα^D842V^* induces a progressive fibrotic adipose tissue phenotype similar to the one in *Spry1KO* mice. These mice also showed increased proliferation and ECM deposition, remarkably similar to *Spry1*KO mice. However, *PDGFRα^D842V^* mutant mice showed fibrosis in multiple adipose depots [12], whereas in *Spry1*KO mice, the progressive fibrosis was most evident in gWAT suggesting unique local environmental cues play a role in gWAT fibrosis in *Spry1*KO mice [12]. Another study showed that PDGFRα+ APCs acquire a fibrogenic phenotype in gWAT of mice fed a high fat diet [13]. Furthermore, these studies showed that PDGFRα+ CD9^high^ cells commit to a fibrogenic lineage whereas PDGFRα+ CD9^low^ cells commit to an adipogenic lineage. Activation of PDGFRα+ promotes a shift towards PDGFRα+ CD9^high^ fibrotic cells [10]. More recently, a study using a mosaic mouse genetic model demonstrated that both PDGFRα and PDGFRβ are cell autonomous inhibitors of adipocyte differentiation [22]. Because SPRY1 is a feedback inhibitor of RTK signalling, SPRY1 deficiency in gWAT may increase basal PDGFR signalling leading to increased SVF proliferation skewing gWAT APC away from adipogenesis and towards a fibrotic phenotype, similar to the *PDGFRα^D842V^* mouse model. Additional studies are required to define the differences in local cues that contribute to fibrosis due to *Spry1* deficiency.

Ex vivo, SVF from *Spry1*KO mice had a greater proliferative response to both PDGF-AA and PDGF-BB. It is noteworthy that SVF cells from both *Spry1*KO and WT mice responded similarly to PDGF-AA and PDGF-BB with regard to ERK activation. This is contrary to what one might expect because SPRY1 deficiency results in enhanced ERK activation in several cellular contexts [23]. However, *Spry1*KO SVF showed enhanced peak AKT activation in response to PDGF-AA and PDGF-BB, and this may contribute to enhanced proliferation. Furthermore, PDGF-AA led to a transient down regulation of SPRY1 expression in wild-type SVF, whereas PDGF-BB led to sustained down regulation of SPRY1. Our data show that SPRY1 is down regulated at the beginning of adipocyte differentiation of WT SVF, but its expression increases as adipocyte marker genes are expressed. We also show that pre-treatment of wild type SVF with PDGF-BB suppresses adipocyte differentiation, whereas PDGF-AA had little effect on adipocyte differentiation. Together, our data suggest that SPRY1 is dynamically expressed in SVF cells and this expression is controlled by environmental cues such as PDGF or cholesterol. Because SPRY1 is up regulated as adipocyte differentiation proceeds, it may act as a break on the APC differentiation programme. This idea is supported by the observation that *Spry1* deficiency results in higher levels of adipocyte differentiation proteins and greater oil red O staining. The notion that environmental cues, in addition to reduced Spry1 levels contributes to fibrosis in *Spry1*KO mice, is supported by data showing that PDGF-BB results in sustained down regulation of SPRY1, and PDGF-BB pretreatment of SVF inhibits adipocyte differentiation suggesting PDGF-BB may drive at least a subset of APC towards a fibroblastic lineage. Data that show that PDGF-AA and PDGF-BB differentially down regulate SPRY1 suggests a possible divergence or specificity of signalling by PDGF-AA and PDGF-BB which may also impact APC cell fate. Additional studies are required to fully elucidate the mechanism by which SPRY1 regulates APC cell fate.

Gonadal WAT fibrosis due to *Spry1* deficiency is of particular interest because a recent study showed that 4 SNPs had a significant association with obesity-related traits and osteoporosis in humans [24]. In another study, weight loss interventions were shown to induce expression of SPRY1 in human APC [18]. Furthermore, SPRY1 expression was low in proliferating human APC but increased upon adipocyte differentiation, and knockdown of human SPRY1 in vitro inhibited differentiation of APCs [10]. Another study using APC from human subcutaneous adipose tissue show that knockdown of SPRY1 in APCs resulted in APCs acquiring a senescence-associated secretory phenotype [25]. This phenotype included induction of senescence inducing p53 and p21^cip^ expression and expression and release of chemokines IL-8 and CXCL1. These data differ somewhat from the data presented here. One possibility is that the human APC were derived from subcutaneous adipose tissue, whereas our studies used gWAT and iWAT from mice. Additional study is needed to resolve these differences.

In summary, our studies show that *Spry1* deficiency in mice results in increase proliferation of gWAT SVF cells with progressive fibrosis. Ex vivo, SPRY1 deficiency results in increased proliferation and differentiation of gWAT SVF cells. Further studies will be required to determine whether the phenotype of gWAT in SPRY1 deficient mice results in metabolic dysfunction as these mice age. Our results, combined with recent studies from other laboratories, demonstrate the importance of SPRY1 in the adipogenic process and suggest possible future strategies to treat obesity and its attendant co-morbidities

## 4. Materials and methods

### 4.1. Animals

All procedures involving animals were approved by the Maine Medical Center Institutional Animal Care and Use Committee (IACUC), and conducted in compliance with ethical and safe research practices. *Spry1LacZ (Spry1*KO) or wild type (WT) mice on an FVB background were from the Mouse Mutant Regional Resource Center (UC, Davis) [15]. Spry1LacZ mice were established with the complete *Spry1 ORF* replaced by the *LacZ* gene that functions as a reporter of *Spry1* promoter activity. Genotyping was performed by PCR using genomic DNA prepared from tails. Knockout of *Spry1* in *Spry1LacZ* mice was verified by immunoblotting from isolated SVF cells or tissues. For phenotype analysis, littermates S*pry1KO* mice and WT controls were used. For SVF ex vivo analyses, mice at same age were used. All animals were fed on a standard chow diet (2018 Teklad global 18% rodent diet, Envigo).

### 4.2. X-Gal staining, histological, and RNAscope analysis

For morphological analysis, adipose tissues were thoroughly fixed in 10% formalin, paraffin-embedded, sectioned and stained with haematoxylin and eosin (H&E). Immunohistochemistry assays were performed using VECTASTAIN ABC kit (Vector Laboratories). Co-immunofluorescence staining was performed by incubation with rabbit anti-Ki67 (Cell Marque Tissue Diagnostics) or anti-PDGFRα (Cell Signalling Technology) followed by FITC-anti-rabbit IgG in combination with Cy3-anti-SMA antibody (Sigma). For x-Gal (5-Bromo-4-chloro-3-indolyl beta-D-galactopyranoside, Sigma) staining, adipose tissues were fixed using 10% neutral buffered formalin for 1 h, whole-mount x-Gal stained, paraffin-embedded, sectioned, and then nuclear fast red counterstained. For RNAscope, adipose tissues were fixed in 10% formalin, cryo-embedded, and then sectioned at 20 to 30 microns for histological analysis. In situ RNAscope assays for *Pdgfra* and *Spry1* were performed according to RNAscope fluorescent multiplex assay instructions (ACDBio). Images were captured using Canon EOS camera and remote imaging software (Canon), or a Leica SP8 confocal microscope.

### 4.3. Isolation, culture and adipogenic differentiation of APCs

SVF cells were isolated as previously reported [26]. Briefly, mice were euthanized by overdose with isoflurane, and then perfused with PBS. Brown adipose tissue (BAT), inguinal WAT (iWAT) and gonadal WAT (gWAT) were collected, cut into 1 mm pieces, and digested with 1 mg/ml type I collagenase, 2 mg/ml dispase (Gibco), 10 mM CaCl_2_ in HBSS for 1 h at 37°C with constant shaking. After inactivating collagenase with 10% foetal bovine serum (FBS) in α-MEM (Corning) the cell suspension was filtered through 70-μM nylon mesh (BD Biosciences) followed by centrifugation at 1,200 rpm for 5 min to separate mature adipocytes (floating fatty cake) from stromal vascular fraction (SVF) cells (the pellet). SVF cells were then resuspended in RBC lysing buffer (Sigma) for 8 min to remove red blood cells. SVF cells were cultured in 10% FBS α-MEM medium. SVF cells from iWAT or gWAT were used at passage 1 to minimize the effect of cell clumps of primary cultures for signalling pathways analysis. SVF cells from gWAT were directly counted and seeded into plates in growth medium with or without PDGFAA, PDGFBB or FGF2 for 24 h, then subjected to adipogenesis analysis. Cells were differentiated into adipocytes with adipogenic induction medium composed of 10% FBS α-MEM containing 0.15 U/ml insulin, 2 nM 3,3ʹ,5-Triiodo-L-thyronine (T3) (Sigma), 10 nM dexamethasone (Sigma), 10 nM hydrocortisone (Sigma), 500 μM 3-Isobutyl-1-methylxanthine (IBMX) (Sigma), 1 μM rosiglitazone (Sigma) for 48 h, followed by maintenance medium composed of 10% FBS α-MEM containing 0.15 U/ml insulin for the remainder of the experiment [26]. Oil Red O staining was used to evaluate the accumulation of lipids. Briefly, cells were fixed with 10% formalin for 10 mins at room temperature, washed with PBS. For Oil Red O staining, cells were washed with 60% isopropanol for 5 min, stained with fresh prepared Oil Red O staining solution (mixture of 0.5% Oil Red O in 60% isopropanol stock with ddH2O at the ratio of 3:2 for 10 mins) for 5 mins at room temperature. Excess dye residue was removed by briefly rinsed with 60% isopropanol and then thoroughly rinsed with water. Images was obtained using Cannon EOS 60D under ZEISS microscopy. For quantification, cellular Oil Red O dye was extracted by incubation with 100% isopropanol, and quantified using a Glomax Explorer plate reader (Promega) at 490 nm. After extraction of Oil Red O dye, cells were washed with water, stained with 0.05% crystal violet for 10 min at room temperature, and thoroughly rinsed with water. Cellular crystal violet dye was extracted with 10% acetic acid, and measured at 600 nm.

### 4.4. Cell proliferation assays

SVF cells were seeded into 96-well plates with or without 20 ng/ml PDGF-AA or PDGF-BB in growth medium. Twenty-four hours later, 10ul (5 mg/ml in PBS) MTT (3-(4,5-dimethylthiazol-2-yl)-2,5-diphenyltetrazolium bromide, Sigma) was added into each well and cultured for another 2hrs. Cells were washed with PBS, purple substrate was extracted with 100ul of 10% SDS and absorbance measured using Glomax Explorer plate reader (Promega) at 600 nm.

### 4.5. Quantitative reverse transcription PCR (RT-qPCR) or RT-PCR and immunoblotting

Total RNA from adipose tissues, fractionated fatty cakes, SVF or cultured cells was extracted using either RNeasy lipid tissue mini kit or RNeasy plus mini kit (Qiagen) according to the manufacturers protocol. RNA quality and quantity were evaluated using agarose gel electrophoresis and Nanodrop spectrophotometer (Thermo Fisher), respectively. cDNA was generated using the ProtoScript First-Strand cDNA Synthesis kit (Bio-Rad), and qPCR analysis was performed using AzuraQuant Green Fast qPCR master mix (Azura) using CFX Connect Real-Time system (BIO-RAD) with one step program: 95°C for 10 min followed by 40 cycles of 95°C for 15 s and 60°C for 30 s. Expression of target gene was normalized to housekeeping gene *Cypa* or *Gapdh*, and relative mRNA level was estimated using the δδCt approach. Results represented on a log2 scale form (fold induction equals 2^(-δδCt)). The sequence of primers is shown in Table 1. For protein assays, adipose tissue fractions or cells were lysed in 2X SDS sample buffer or in HNTG buffer [50 mM HEPES, pH 7.4, 150 mM NaCl, 10% (vol/vol) glycerol, 1.5 mM MgCl_2_, 1 mM EGTA] containing protease inhibitor cocktail (Roche), phosphatase inhibitors and 0.2% Triton X-100. Lysates were subjected to SDS-PAGE and immunoblot analysis using antibodies against PPARγ, SPRY1, pERK, ERK1/2, pAKT, AKT (Cell Signalling Technology), SMA, and tubulin (Sigma). Immunoblotting was performed first using antibodies against target proteins, developed and thoroughly washed followed by immunoblotting for one of the housekeeping proteins including GAPDH, beta-Actin and beta-Tubulin. The quantification of target protein was calculated by normalizing to internal housekeeping protein. The phosphorylated proteins and their corresponding total protein were immunoblotted separately using differently blots and followed by immunoblotting for housekeeping protein whenever possible. Quantification of immunoblotting results was performed using ImageJ.

**Table 1.** RT-qPCR or RT-PCR primer sequences designed using NCBI primer designing tool

Symbol	Primers	NCBI sequence
*Spry1*	Forward: 5ʹ-ATGATTGTCTTTGGTTGGGCTG-3’	NM_011896.1
	Reverse: 5ʹ-CTGGTGGGTGTCAACATTGTC-3’	
*SMAa*	Forward: 5ʹ-CGCTGTCAGGAACCCTGAGA-3’	NM_007392.2
	Reverse: 5ʹ-CGA AGC CGG CCT TAC AGA-3	
*Fibronectin*	Forward: 5ʹ-CAGCAGTATGGCCACAGAGA-3’	NM_010233.1
	Reverse: 5ʹ-AAAGCTGCTGGCTGTGATTT-3’	
*Mmp2*	Forward: 5ʹ-CTGATAACCTGGATGCCGTCGTG-3’	NM_008610.2
	Reverse: 5ʹ-AAAGCTGCTGGCTGTGATTT-3’	
*Pparg*	Forward: 5ʹ-GGGGTGATGTGTTTGAACTTG-3’	NM_011146.3
	Reverse: 5ʹ-CAGGAAAGACAACAGACAAAT-3’	
*FABP4*	Forward: 5ʹ-GATGCCTTTGTGGGAACCT-3’	NM_024406.2
	Reverse: 5ʹ-CTGTCGTCTGCGGTGATTT-3’	
*Adiponectin*	Forward: 5ʹ-GAACTTGTGCAGGTTGGATG-3’	NM_009605.4
	Reverse: 5ʹ-TGCATCTCCTTTCTCTCCCT-3’	
*Leptin*	Forward: 5ʹ-CCTCATCAAGACCATTGTCACC-3’	NM_008493.3
	Reverse: 5ʹ-TCTCCAGGTCATTGGCTATCTG-3’	
*Ucp-1*	Forward: 5ʹ-CGATGTCCATGTACACCAAGGA-3’	NM_009463.3
	Reverse: 5ʹ-ACCCGAGTCGCAGAAAAGAAG-3’	
*MCP-1 (CCL2)*	Forward: 5ʹ-AGCACCAGCCAACTCTCAC-3’	NM_011333.3
	Reverse: 5ʹ-TCTGGACCCATTCCTTCTTG-3’	
*Col1a1*	Forward: 5ʹ-GTCTCCTGGTATTGCTGGT-3’	NM_007742.3
	Reverse: 5ʹ-GGCTCCTCGTTTTCCTTCTT-3’	
*F4/80*	Forward: 5ʹ-CTTTGGCTATGGGCTTCCAGTC-3’	NM_010130.4
	Reverse: 5ʹ-GCAAGGAGGACAGAGTTTATCGTG-3’	
*Cyclophilin*	Forward: 5ʹ-CCACCGTGTTCTTCGACAT-3’	NC_008907.2
	Reverse: 5ʹ-CAGTGCTCAGAGCTCGAAAG-3’	
*Gapdh*	Forward: 5ʹ-ACACATTGGGGGTAGGAACA-3’	BC023196.2
	Reverse: 5ʹ-AACTTTGGCATTGTGGAAGG-3’	

### 4.6. Statistical analysis

GraphPad Prism 7.0 was used to perform the statistical analyses. Data were analysed by using either 1-way or 2-way ANOVA. When justified, multiple-comparisons tests were used to determine the p value. In some cases, student t-test was used for power analysis. Differences were considered significant at p < 0.05, and all results are reported as means ±SEMs.

## Data Availability

Data are available upon request from the authors.
